# An Open Hardware Design for Internet of Things Power Quality and Energy Saving Solutions

**DOI:** 10.3390/s19030627

**Published:** 2019-02-01

**Authors:** Eduardo Viciana, Alfredo Alcayde, Francisco G. Montoya, Raul Baños, Francisco M. Arrabal-Campos, Francisco Manzano-Agugliaro

**Affiliations:** Department of Engineering, University of Almeria, 04120 Almeria, Spain; edu.vg82@gmail.com (E.V.); aalcayde@ual.es (A.A.); rbanos@ual.es (R.B.); fmarrabal@ual.es (F.M.A.-C.); fmanzano@ual.es (F.M.-A.)

**Keywords:** smart meter, low cost, network analyser, electricity, power quality, energy metering

## Abstract

An important challenge for our society is the transformation of traditional power systems to a decentralized model based on renewable energy sources. In this new scenario, advanced devices are needed for real-time monitoring and control of the energy flow and power quality (PQ). Ideally, the data collected by Internet of Thing (IoT) sensors should be shared to central cloud systems for online and off-line analysis. In this paper openZmeter (oZm) is presented as an advanced low-cost and open-source hardware device for high-precision energy and power quality measurement in low-voltage power systems. An analog front end (AFE) stage is designed and developed for the acquisition, conditioning, and processing of power signals. This AFE can be stacked on available quadcore embedded ARM boards. The proposed hardware is capable of adapting voltage signals up to 800 V AC/DC and currents up to thousands of amperes using different probes. The oZm device is described as a fully autonomous open-source system for the computation and visualization of PQ events and consumed/generated energy, along with full details of its hardware implementation. It also has the ability to send data to central cloud management systems. Given the small size of the hardware design and considering that it allows measurements under a wide range of operating conditions, oZm can be used both as bulk metering or as metering/submetering device for individual appliances. The design is released as open hardware and therefore is presented to the community as a powerful tool for general usage.

## 1. Introduction

As electricity distribution grids are being developed, the need for metering has increased. In 1977, Paraskevakos [1] presented the first automatic and commercialized remote meter. Nevertheless, the concept of remote and smart metering was not considered for many years. Many concerns in recent years have arisen, including climate change, trends in the electricity markets, efficiency, and the promotion of renewable energy resources. In addition, other active agents in power systems are promoting distributed grids with distributed storage. This drastic change requires an evolution in the actual electricity model [2].

The core of the smart grid concept resides in an electricity grid model able to treat different energy sources in an efficient and decentralised manner. The capability for a new metering system or smart metering will manage this smart grid. Smart metering or an intelligent metering system is defined as “an electronic system that can measure energy consumption, providing more information than a conventional meter, and can transmit and receive data using a form of electronic communication.” This definition was established by the European Parliament in its 2012/27/EC directive [3]. The implementation of the energy efficiency directive can be found in [4].

This implementation of the energy efficiency directive is a consequence of the transition of power grids from centralised generation to distributed generation, motivated by global trends in environmentally friendly power generation systems. This is possible if the real state of the grids is known. This requires fine-grained knowledge of the state itself. The concept of the smart grid allows for the delivery of electricity in a controlled system.

A smart grid is an electric grid that joins points of generation to points of consumption, which recognize the real state of the net. Since a smart grid is conceived to deliver electricity, the consequence is the emergence of new and developing technologies in power generation, transmission, and distribution. This can be considered as a class of technology driven by recent advances in renewable energy sources and modern communications and computation technologies [5]. The electric grid is more than a generation and transmission infrastructure. It is an ecosystem with assets from government, providers, manufacturers, and owners. This technology set up three foundations: advanced renewable energy sources, control systems, and computer processing [6]. These advanced technologies must be composed with advanced sensors known as advanced measurement units or multipurpose network analysers and smart meters [7], allowing operators to assess grid stability and provide consumers with better information including automatically reported outages. The system includes relays that sense and recover from faults at a substation automatically, automated feeder switches that reroute power around problems, and batteries that store excess energy and make it available later to the grid to meet customer demand.

Many studies have highlighted the advantages derived from the progressive development of distributed generation systems, smart grids, and microgrids [8,9]. Under this new paradigm, some authors have drawn attention to the importance of developing new sensors and tools for the real-time monitoring and control of the energy flow and power quality. For example, in [10] the residential power consumption is modelled using smart meters and data mining techniques. Other researchers have proposed systems for real-time detection and classification of power quality disturbances in smart grids [11]. These smart metering systems are often integrated with computing and communication technologies to enhance efficiency and reliability of future power systems with renewable energy resources [12]. Further, smart meters include efficient control and processing algorithms [13]. In [14] smart handheld devices were used to perform the real-time monitoring and fuzzy control strategies of an intelligent windowsill system in a smart home. In [15] neural network and swarm intelligence approaches were successfully applied to analyze the dynamic operation and control strategies for a microgrid hybrid power supply systems. Moreover, the system stability has also been considered in by some researchers. In [16] it was proposed a direct building algorithm for microgrid distribution ground fault analysis that consider the network topology changes. The topological characteristics of the microgrid are also considered in [17], where it was proposed an unsymmetrical faults analysis method with hybrid compensation for microgrid distribution systems. In [18] it was proposed an optimization algorithm to manage real-time congestions using all power system capabilities.

Complex networks and the advance of electronic devices have inspired the power quality (PQ) concept [19], that is, the measurement of the amount of disturbance in the electricity supply, which is later assessed. Millions and millions of electronic devices are plugged into the grid, affecting the global grid. Most PQ problems result from grid-connected distributed generations. In addition, nonlinear electronic loads introduce PQ disturbances into the grid because electric car chargers, rectifiers, lighting, switch-mode power supplies, and other power electronic devices are commonly used [20].

PQ disturbances such as swell, sag, interruption, oscillatory transients, flicker, and harmonic distortion are becoming challenging issues for power systems. Modulated power sinusoids have also been detected. Both can manifest as steady-state or transient states. These disturbances may impose penalties on consumers by adding reactive power, redispatch costs, and load curtailment costs. Moreover, these PQ problems can overheat the transformers, damage capacitor banks, and affect sensitive electronic equipment [21]. Owing to the increase of these issues, scientists and engineers have started to study them through significant number of PQ studies [22,23,24,25]. Electric power quality monitoring has become an essential part of these studies [26].

Mitigating actions against power quality disturbances must be taken, and their sources and causes must be identified accurately as well. Power quality disturbances must be detected, recognized, and classified [27,28]. Engineers have to select the appropriate technique for extracting any power quality disturbance in order to recognise and classify them. In recent years, many techniques (apart from Fourier and Short Time Fourier Transform) have been implemented for extracting these features of power quality disturbances, including the S-transform [29] where a new detection and classification method of power quality disturbances based on S-transform, interpolating windowed fast Fourier transform (FFT), and probabilistic neural network (PNN) was proposed; wavelet transform [30] where wavelets and fuzzy support vector machines are used to automated detect and classify power quality (PQ) disturbances; Kalman filters [31] where a practical model describes the behavior of electric arc furnaces in the simulation of power system for power quality issues; Gabor transform and Wigner distribution function [32] where improved Gabor-Wigner transform algorithm is studied and applied to power quality disturbance detection; empirical mode decomposition [33] where performance comparisons of Support Vector Machine (SVM) and different classification method for power quality disturbance classification is presented; compressive sensing (CS) [34] where a new approach for classifying multiple power quality disturbances (PQD) is presented; curvelet [35] where a new scheme based on curvelet transform and support vector machine is proposed; Hilbert transform [36] where a new classification method of transient disturbance based on Hilbert transform and classification trees is proposed; Hilbert-Huang transform [37] where the power quality in the distribution network is considered to characterize power signal disturbances; and hybrid transform-based methods [38] where a sparse method for PQ representation, starting from overcomplete dictionaries, is introduced. These techniques are used as input to a classifier such as support vector machine [39] where a new hybrid algorithm is presented for PQ disturbances detection in electrical power systems with SVM; fuzzy logic [40] where a fuzzy logic based online fault detection and classification of transmission line using Programmable Automation and Control technology based National Instrument Compact Reconfigurable i/o (CRIO) devices is introduced; ensemble technique [33] where performance comparisons of SVM and different classification method like ensemble mode decomposition for power quality disturbance classification is analyzed; deep learning [41] where a deep learning-based method is introduced into the classification of power quality disturbances; artificial neural network [22] where a conjugate gradient back-propagation based artificial neural network for real time power quality assessment is proposed; and maximum likelihood classifier [42] where a newly developed CS, maximum likelihood (ML), and rule-based method for classification of power quality disturbances.

The technology for smart metering is a heterogenous infrastructure that includes many branches of engineering: communication networks, data processing, management, and installation. The minimum infrastructure for this system of smart metering is the electronic device for metering (smart meter), a cloud-based data gathering system, a communication system (normally based on the Internet), and a computational centralised center. A few academic researchers have presented energy meters and power quality analysers, but up to now, most of the installations are monitored by using commercial devices. However, these devices are often expensive and difficult to manage by non-expert users. Therefore, it is a challenge to develop low-cost and high-precision devices for general usage in any type of electrical installation. This is precisely the main contribution of oZm, a small-sized open source, low-cost, precise, and reliable power and electric energy meter aimed to help people to analyze and visualize energy consumption measures complying with international standards such as IEC 61000-4-30 and EN-50160. Herein, the openZmeter (oZm from now on) device is described as a key part of a smart metering infrastructure. This electronic device is a major redesign of the basic prototype in [43]. It is low-cost, single-phase open-hardware, with advanced bidirectional capabilities applied to monitoring electric features and cloud-based data gathering. This smart meter presents a wide range of features, which accomplish the minimum desirable requirements for an electricity smart meter as published in recommendation 2012/148/EU [44]. The smart meter here presented is useful to monitor and prevent distribution network problems. Thanks to the features of oZm, it is possible to receive information about significant events detected in the electrical installations. By monitoring these abnormal situations, e.g., high power consumption or significant power quality disturbances, it would be possible to implement preventive strategies.

The main contribution of this paper is to present a professional low-cost open-source hardware device for power quality and energy metering and submetering for the Internet of Things. The advanced design of oZm ensures reliable and accurate energy consumption and power quality measurements under a wide range of operating conditions in a single handy device. Given its small size and large variety of current probes, it can be easily installed at any electrical installation, i.e., it can be used both as bulk metering or as metering/submetering for individual units or appliances. Unlike other typical low-cost data loggers or meters like those analyzed in [45], oZm records all electrical parameters and events following international standards. Finally, other important contribution of the paper is to present and display a large number of measurements with an user-friendly interface that can be customized according the particular needs.

The rest of the paper is organized as follows: Section 2 presents a detailed overview of the hardware and software of the smart meter design. Section 3 presents an analysis of its usage in a real environment, while the main conclusions are summarized in Section 4. The acronyms used in the text are listed in Table 1 in alphabetic order together with their meaning in full words as found in literature.

## 2. IoT Power Quality and Smart Meter Design

Herein, an open-hardware platform for PQ monitoring and smart energy metering is designed, developed, and manufactured. This electronic device was designed for monitoring PQ events, voltage, and current waveforms (along with other advanced features) following the specifications given by international standards such as IEC 61000-4-30 and EN-50160. The system for PQ monitoring and smart metering can be considered an Internet-based power quality monitoring and smart metering system because of its accurate probes and embedded server that manage all PQ and energy information over the Internet or a local network. Raw and processed values can be accessed locally from an internal database through a web application or uploaded to a central server in the cloud for massive deployment.

Currently, single-phase hardware has been successfully built, and a three-phase version is already in the prototype stage. Because of its characteristics, this single-phase version is mainly used in domestic environments, but there is no limit to using it elsewhere if the current is below 35 A RMS because the Hall effect current sensor used is limited to a 50-A peak value. However, the system is able to accept any other current probe (split-core, Rogowski, etc.) if the voltage output is between ±1.25 V. All components are isolated from the main voltage in order to allow safe operation of the system. For industrial applications, the three-phase version is recommended. In this case, high-accuracy current clamps or Rogowski coils should be used to sense the current.

The core of the Analog Front End (AFE) is the STM32F373 from the STM32 microcontroller family, which combines high performance, real-time capabilities, digital signal processing, and low-power operation. The STM32F373 is the smallest micro in the family with onboard 16 bit Analog Digital Converter (ADC). In this case we looked for the highest precision without increasing too much the number of components, thus avoiding the use of external ADCs. Also, the manufacturer STMicroelectronics classifies this model as "High precision line" for metering purposes. The onboard ADC is used for signal sampling and conditioning. The conditioned signal is filtered and limited. Then, a correction for gain and offset is applied. This ADC can sample values up to 16 kHz per channel.

The current and voltage signals converted are preprocessed by the digital signal processing (DSP) module of the STM32 using custom signal algorithms designed by the authors. Afterward, the output is sent to a poweful embedded ARM board for final processing. Complex calculations such as FFT, zero crossing, and RMS values are then executed by a Linux core (the host), where a daemon service is run in an endless loop in real time. This real-time strategy allows to implement priorities for threads and queues, so that critical processes are treated with a high priority and less critical processes have a low priority.

The software and hardware repository is publicly available and can be accesed at https://gitlab.com/zredalmeria/openZmeter.

### 2.1. Hardware Structure of oZm

Advanced electronics such as microprocessors or field programmable gate array (FPGA) devices are commonly used for PQ monitoring and energy smart metering. Every choice has its own advantages and drawbacks. Recently, the use of highly efficient and low-cost ARM boards has been demonstrated to be a real option when coupled to other microcontrollers. In our case, smart metering and PQ monitoring are accomplished using a Linux ARM board and AFE managed by an STM32 microcontroller.

In terms of cost, this approach is the most efficient. Within the evolution of hardware, it is possible to find low-cost DSP devices that can work at high speeds with more integration and more features at a cheaper cost. Moreover, the ARM Linux controller is reliable, efficient, and low-cost. It has also been proven to be much more flexible than any other device thanks to its software repositories and packages.

**Figure 1:**
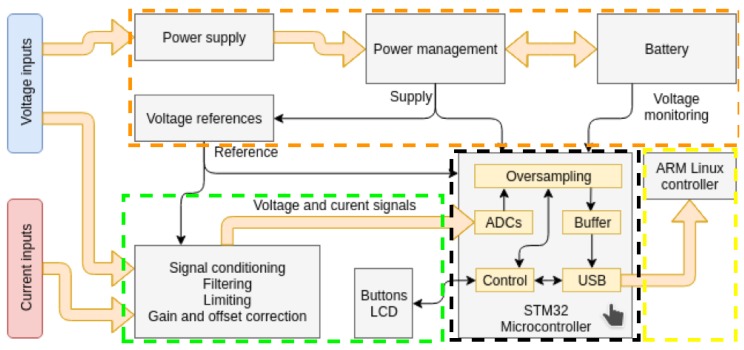
openZmeter (oZm) hardware block diagram. Block for signal acquisition coloured in green, power supply and management elements coloured in orange, STM32 microcontroller in black, and advanced RISC machine (ARM) Linux board controller in yellow.

Figure 1 shows a block diagram for the oZm hardware, where four main blocks can be identified. The first one (coloured in green) is the signal acquisition stage for current and voltage. An accurate resistor divider is used to sense and reduce the main voltage to a low level around a few volts. Filtering and correction are applied using a low-pass filter in order to avoid aliasing effects. The current is sensed by means of a Hall effect integrated circuit (IC) from Infineon. The second block (coloured in orange) is composed of a power supply and management elements, including the Li-Po battery.

The third block (coloured in black) is an STM32 microcontroller that handles all acquired signals, i.e., sampling, buffering, and USB communication with the fourth block (coloured in yellow), which is the ARM Linux board controller. Here, major processing tasks are executed along with database storage, web server functions, and web visualization.

**Figure 2:**
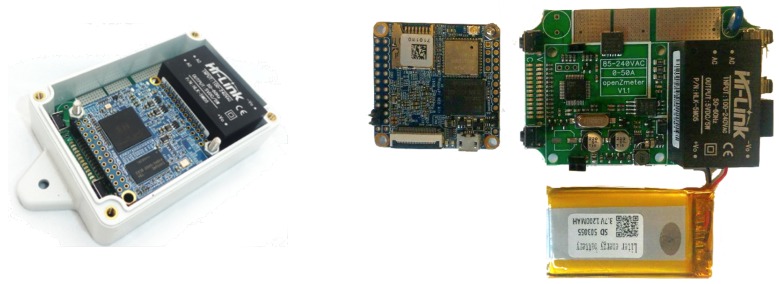
oZm hardware elements. (**Left**): full system including plastic enclosure. (**Right**): components side by side with ARM nanopi Air, analog front end (AFE), and Li-Po battery.

Figure 2 presents some views of the hardware. A general view is on the left side, and a detailed component view is on the right. The ARM board, Li-Po battery, and AFE can be seen.

The voltage input is protected against overvoltage and transients. Protection is realized by means of a voltage limiter using a varistor. To protect against overcurrent, a fuse is also installed. Moreover, the resistance of the fuse limits the high current required during the connection because of the charging of capacitors from the main voltage. A jumper selects the operating mode, which must be provided with the appropriate connection. The oZm is single phase and has an integrated Hall-effect sensor.

**Figure 3:**
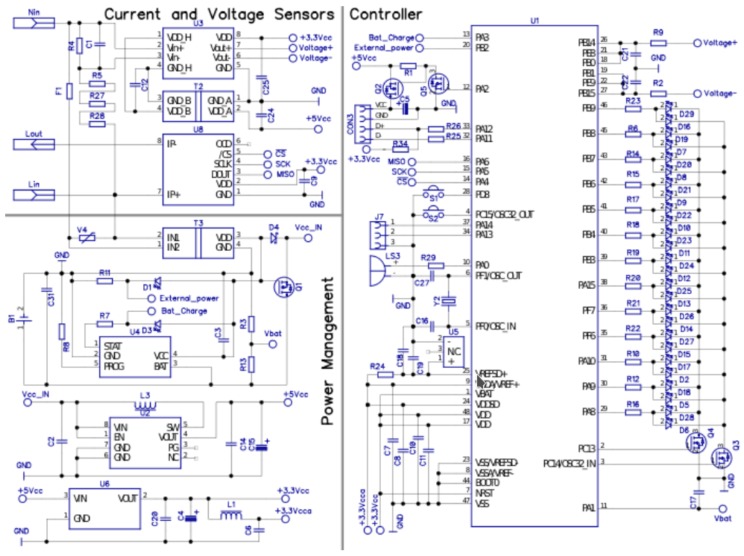
Electronic scheme of single-phase device. Controller, current/voltage sensor, and power management are shown.

**Figure 4:**
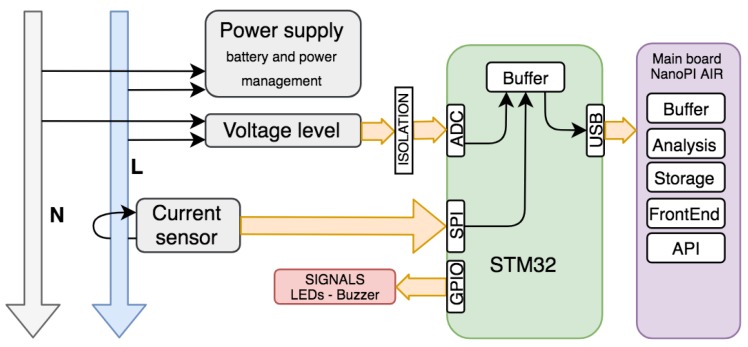
Flowchart for the electronic scheme.

**Figure 5:**
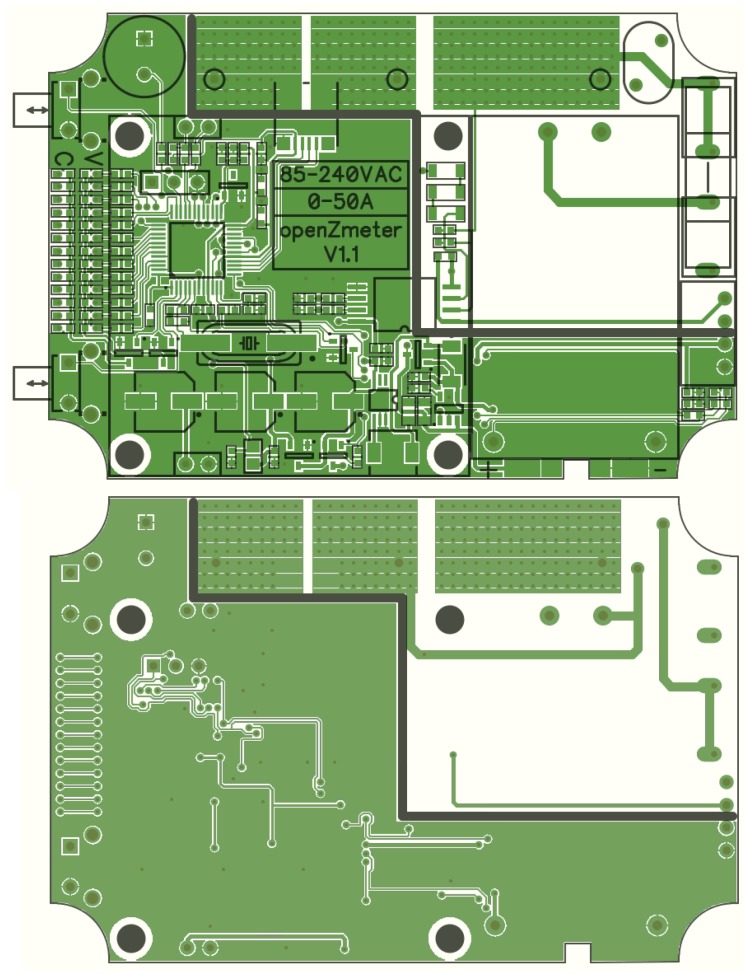
Printed circuit board (PCB) silkscreening of oZm board.

In Figure 3, the proposed electronic scheme is presented along with a flowchart in Figure 4. It can be seen that the current flows in the oZm through a Hall-effect sensor integrated in the PCB (Infineon TLI4970). Figure 5 shows a detailed silkscreen of the PCB made with opensource software Kicad [46]. For safety reasons, the PCB tracks are sized for the maximum current of 50 A allowed by the sensor. No further processing is required on this channel thanks to the serial SPI connection to our STM32. The samples of the voltage channel are obtained through the isolation amplifier (U3) ACPL-C79 model from Broadcom. This device provides the necessary isolation from the mains voltage.

To obtain the input signal, a phase-to-neutral resistive divider is used. This reduces the voltage to ±200 mV for an input range of approx. ±300 V. This output is then redirected to a Broadcom ACPL-C79 isolation amplifier (input signal of ±200 mV), which converts to a differential output signal of ±2.5 V. The splitter incorporates an RC filter that attenuates frequencies above 16 kHz. The ACPL-C79 incorporates a ΣΔ modulator that converts the input voltage into a train of pulses that are transmitted optically to a ΣΔ converter that restores the signal to the output range set internally to ±2.5 V. The optical coupling provides the required insulation.

This topology requires a power supply for the part connected to the network that is isolated from the power supply of the module. This function is performed by a DC/DC converter (Recom RFM-0505S). The obtained signal from the isolation amplifier is injected into the three ADC channels of the STM32 after passing through an RC filter that attenuates out-of-range frequencies, from where the sample conversion can be coordinated. Conversion is performed with 16-bit accuracy and a maximum speed of 16 kHz per ADC channel. The STM32 has a great flexibility in the capture process, allowing for the use of only one connected channel and for 16-kHz sampling or sequencing of the three channels, thus increasing the sampling frequency. These functions, as well as the synchronization of the voltage and current samples, are performed by the firmware running on the STM32.

The voltage is conditioned to the appropriate level. An AC/DC converter module gives a stable 5-V output in the range of 85 to 265 VAC for the single-phase version. The galvanic isolation is set between the network and the PCB components. Four blocks are implemented in the power supply:

- Input rectifier: Rectifier diodes convert AC to DC, which must withstand at least twice the mains voltage. The device can be used, in safety conditions, up to 1000 V.
- Input filter: To prevent noise sources from spreading to the mains, and to stabilize the rectified voltage.
- Buck converter: Reduces the voltage to 22 V in order to offer up to 4.5 W.
- Secondary buck converter: The IC used has some limitations. The voltage must be set at 5 V, and this voltage must be stable. A second buck converter is used with the MCP16312 to obtain a stable voltage at 5 V, with low ripple and up to 1 A of current.

A battery is also integrated in the power chain circuitry. oZm is manufactured to remain active even when there is no voltage in the inputs, or when the power supply is not in the operating range. A lithium-ion battery with built-in protection is included. The battery provides a voltage between 3.5 V and 4.2 V, incorporating the necessary electronics to avoid overloads and accelerated discharges. The PCB incorporates a charge controller to store energy while connected to the mains. The load manager is the MCP73832 module, which limits the maximum load current to about 100 mA.

The components of the oZm require 5 V to operate correctly. A DC/DC boost converter (MCP1642) is necessary to adjust the voltage from the battery (voltage between 3.5 V and 4.2 V) and some oscillations in the power supply. Therefore, a final conversion is performed to adjust the voltage to 5 V. The circuit designed uses a typical layout offered by the manufacturer. The selection of one input or another (battery or external power supply) is made by means of a MOSFET, which connects the battery to the input of the converter when there is no voltage in the input converter.

### 2.2. Real-Time PQ Monitoring and Smart Metering Software

The oZm software consists of an embedded ARM unit, where a daemon service runs in an endless loop. The acquisition is performed in real time using an STM32 microprocessor and buffers the ARM unit running a real-time kernel (Linux RT). The daemon service is mainly coded in C++ to obtain a robust and reliable system. Being an Internet-based sensor, it incorporates a wireless module (Ampak AP6212) with WiFi: 802.11b/g/n and Bluetooth: 4.0 dual mode. This makes it possible to connect to the device and monitor the PQ parameters using a web browser with https secure encryption layer. The real-time electric measurement list is as follows:

- RMS values for voltage and current
- Active power, reactive power, and apparent power
- Phase between current and voltage
- Power factor
- Harmonics up to 50th for current and voltage
- Active energy and reactive energy
- Frequency
- Voltage events such swell, sags/dips, and interruptions

Based on the above list of measurements, the service can present clear visualizations based on the following features:

- Energy consumption and generation in four quadrants
- Operation in accordance with international standards IEC 61000-4-30 and EN-50160
- Aggregation for the voltage channel of 3 s, 1 min, 10 min, and 1 h as extra aggregation for energy metering purposes
- Alert system and event management (ITIC/CBEMA, frequency, etc.)
- Cutting-edge HTML, Javascript, and CSS3 technologies for a user-friendly dashboard interface
- API for third-party integration using JSON

**Figure 6:**
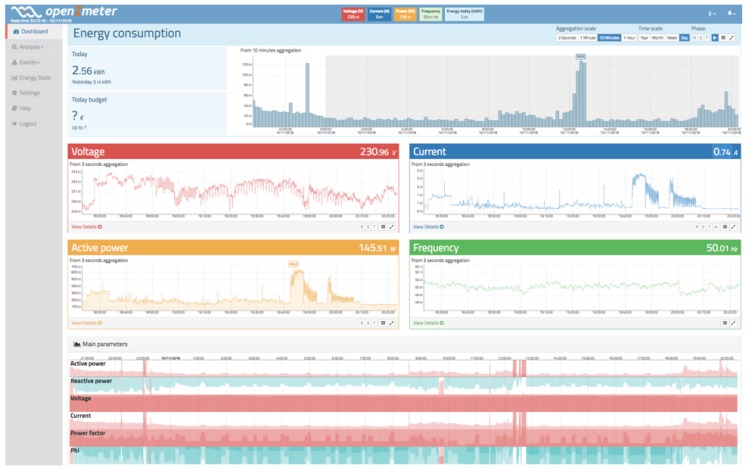
Dashboard of real-time power quality (PQ) monitoring and smart-metering software.

Figure 6 shows a main view of the real-time PQ monitoring and smart metering software.

The parameters are displayed in real time and are presented using an intuitive dashboard, where the data are refreshed dynamically using cutting-edge web technologies such as HTML5, CSS3, and Javascript. Five blocks are plotted for active energy consumption, RMS voltage, RMS current, active power, and grid frequency. Each plot can be maximized for better inspection. All blocks are plotted using a 3 s aggregation interval over the previous 2 h to obtain fine-grained resolution. In addition, the energy block has a more advanced selector in order to choose other resolutions or aggregations. All blocks except for the energy block are synchronized to display information for the same time frame. The rest of the web interface is divided into six sections: voltage, current, power, frequency, voltage events, and energy stats.

Per IEC61000-4-30, the preferred time interval window is 10 cycles of the voltage waveform, i.e., 200 ms for a perfect 50 Hz European grid signal. Using this time interval allows harmonic calculations to be synchronous to all other values such as RMS and Total Harmonic Distortion (THD). In the case of a 60 Hz grid, 200 ms is equivalent to 12 cycles.

Harmonics are calculated using an adapted version of the well-known and stable open-source FFTW3 implementation [47] up to the 50th order for both voltage and current. The data are stored in the local database each 200 ms, and it is possible to retrieve aggregated data using custom methods implemented in the software. Interharmonics components were minimized thanks to the implemented digital filters, which remove artifacts and allow for precise frequency measurement based on zero-crossing techniques.

External time synchronization was implemented to achieve accurate timestamps by means of network time protocol (NTP) time servers. Accuracy is specified at ±20 ms for 50 Hz and ±16.7 ms for 60 Hz instruments with a 10 min interval sync to clock and a 2 h interval sync to clock. To ensure compliance with standard specifications, an efficient NTP implementation was deployed using the Chrony package for Linux [48].

## 3. Application and Results

**Figure 7:**
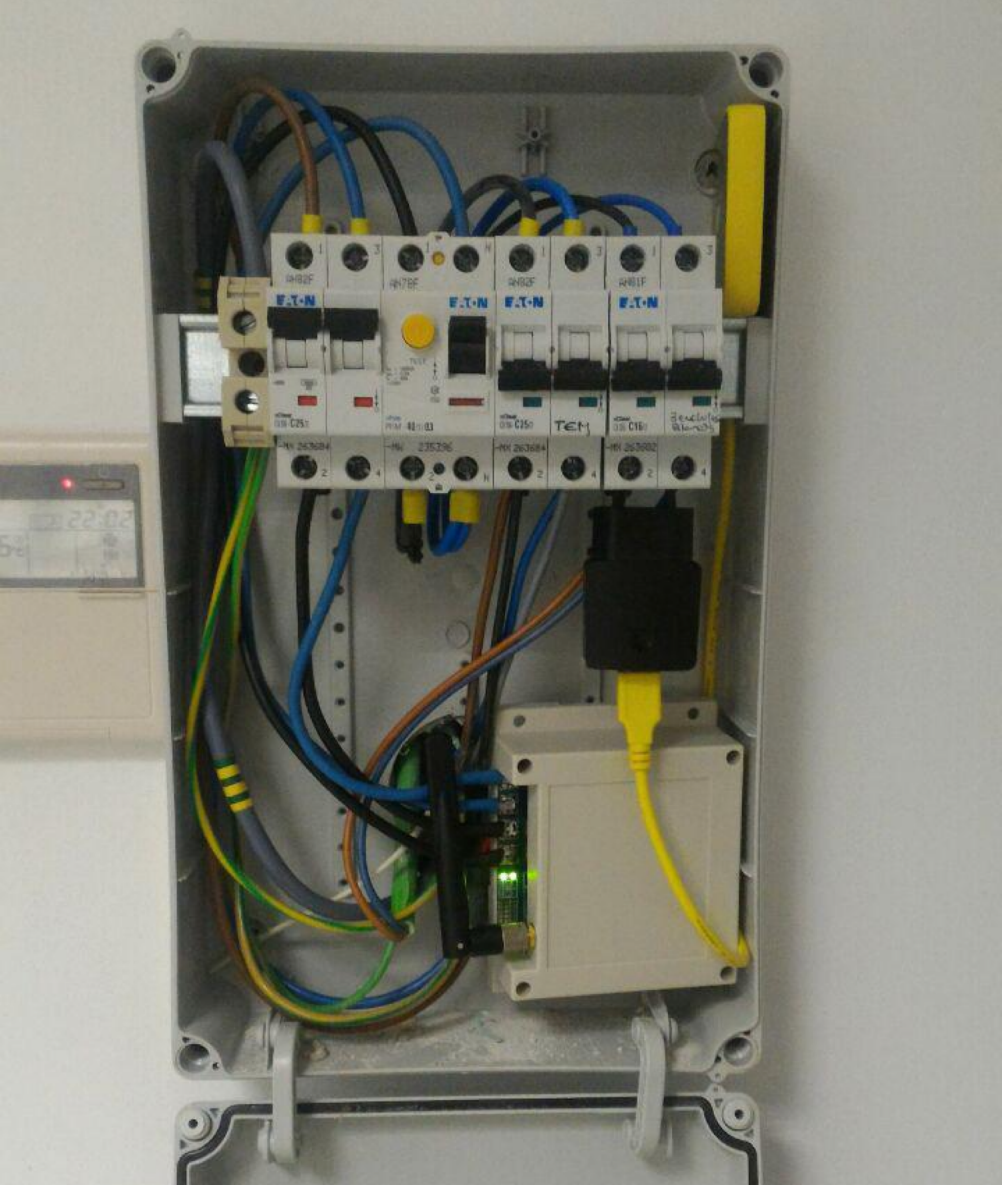
oZm installed in facilities of the University of Almería.

The oZm was installed and tested at some laboratories of the University of Almeria (UAL) and, additionally, in some houses for domestic use. The acquired data was recorded and stored in the oZm, which is accessible using the campus network at any time. The installation of the device was straightforward. Figure 7 shows the final setup of an oZm installed in one of the laboratories at the University of Almeria.

### 3.1. Voltage Analysis

A real-time measurement of the system RMS voltage can be found in the voltage analysis view. Using the selectors, an aggregation level between 3 s and 1 h can be chosen. In this way, the evolution of the voltage for a specific time scale can be displayed in greater or lesser detail. The system can store values of several years depending on the size of the memory chosen on the ARM Nanopi Air board (8-GB standard). The data can be displayed on a daily, weekly, monthly, or annual time scale. The data can be viewed in full screen and can also be downloaded in CSV format for external analysis with specific software. The maximum, minimum, and mean values for the selected time range are shown to the left of the RMS chart.

**Figure 8:**
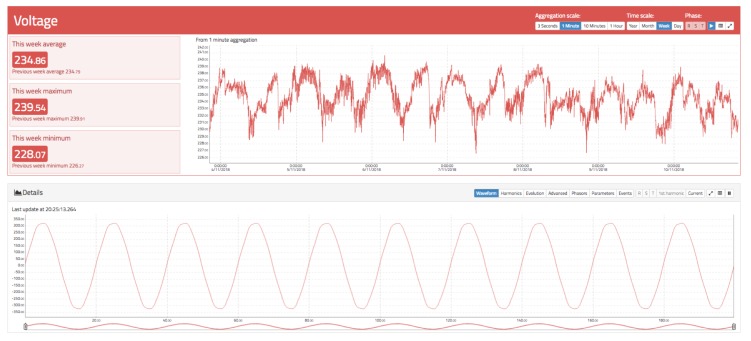
Voltage view of oZm.

**Figure 9:**
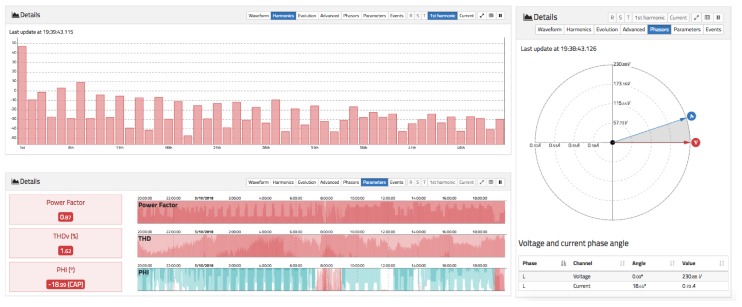
Advanced views of voltage tab (1). (**Top left**): real-time harmonic content for voltage. (**Bottom left**): 24 h of power factor, total harmonic distortion voltage (THDv), and phase angle. (**Right**): phasor representation for voltage and current.

**Figure 10:**
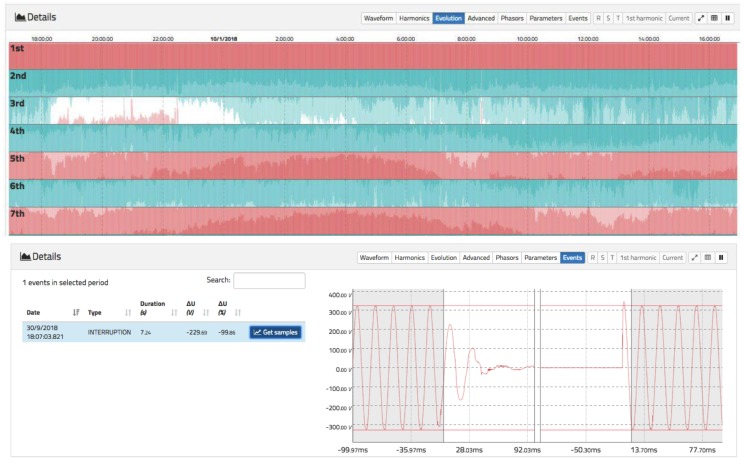
Advanced views of voltage tab (2). (**Top**): harmonic evolution. Darker red colours indicate higher values than darker blue. (**Bottom**): events view with pre- and post-event waveforms.

Below the RMS chart, a detailed chart is available to display the waveform in real time (similar to an oscilloscope) for the last 10 cycles. Harmonics can also be displayed in real time, and the daily evolution of each harmonic from the fundamental to order 50. There is also a visualization that represents a phasor chart for voltage and current. Finally, the events tab can be accessed, where different voltage events occurring in the selected time span can be displayed. These details can be seen in Figure 8, Figure 9 and Figure 10.

Figure 8 shows the RMS voltage variation during a day for a 1 min aggregation scale. It can be observed that the voltage goes up and down during the day, causing an increase as the evening goes by, reaching a minimum around 6:30 p.m. and a maximum around 4:00 a.m. In the same way, the notches produced by voltage drops in the supply line owing to the connection and disconnection of loads in the installation itself can be clearly seen. If the selected aggregation level is set to a lower value, the RMS signal will contain more noise that captures more details. In the same Figure 8 below, the waveform for the supply voltage can be seen. It has a near sinusoidal shape but has some distortions owing to the presence of harmonics.

Figure 9 shows the harmonic spectral content for the signal in Figure 8. It can be seen that the odd harmonics (typical of commercial supplies) have a higher value than the even harmonics. In particular, the 3rd, 5th, and 7th harmonics have the highest value. In the lower part of Figure 9, the evolution of the values of the power factor, Total Harmonic Distortion for voltage (THDv), and phase angle between the voltage and fundamental current for the last 24 h is shown. Darker colours indicate higher values. For the phase-angle chart, positive (red) and negative (blue) values are shown, so it is possible to visualize the inductive or capacitive nature of the load. Finally, on the right of Figure 9, there is a phasor chart of current and voltage, where the capacitive nature of the load is appreciated since the current overtakes the voltage. The values of this phasor chart are obtained by applying an FFT and plotting the module and phase angle between the fundamental components of the voltage and current.

Figure 10 has a similar display as that shown in Figure 9. The evolution of the last 24 h for harmonics up to order 50 can be examined, although owing to space constraints only up to the 7th harmonic is shown. Dark colours in red indicate high values for the magnitude of the value, while dark values in blue indicate low values. Note that even harmonics always exhibit a small value compared to odd harmonics. In addition, it is interesting to observe how the 3rd harmonic varies between large and small values depending on the time of day. In particular, it has higher values at sunset between 6:00 p.m. and 11:00 p.m. than the rest of the day.

Figure 10 details the view of voltage events. A shows the different events that the system can handle: dip, swell, rapid voltage change (RVC), and interruption. In this case, an interruption of 7.24 s is shown. On the right, it is possible to visualize the precise moment before and after the interruption.

### 3.2. Current Analysis

**Figure 11:**
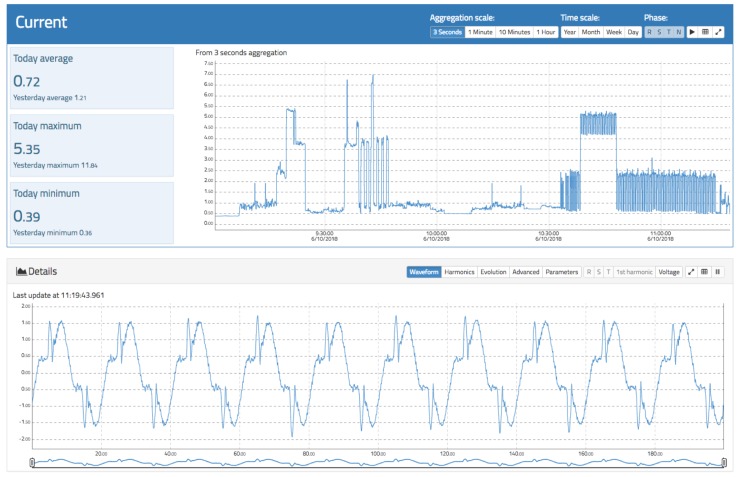
View of root mean square (RMS) and waveform current in oZm.

**Figure 12:**
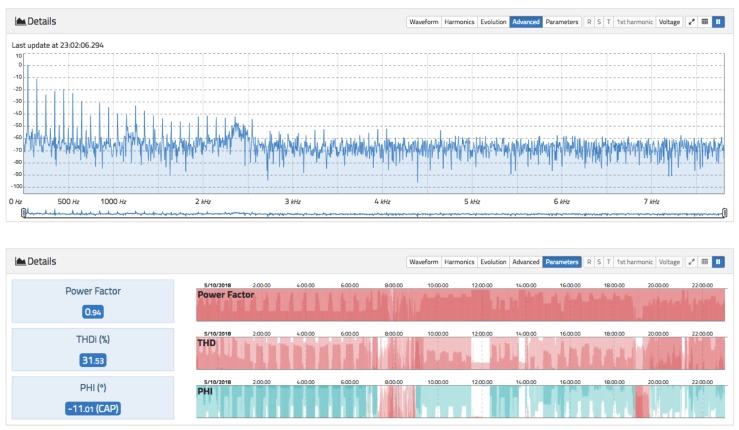
Top: current spectrum. Bottom: advanced parameters THDi, power factor, and phase angle for current.

The current analysis view follows the same approach as the voltage view. Here, a main chart showing the evolution of the RMS current for different aggregation levels and time spans in real time is presented. The data can be downloaded in CSV format. It is also possible to visualize the waveform, harmonics evolution, and advanced parameters such as Total Harmonic Distortion for current (THDi) and power factor. Figure 11 and Figure 12 show the relevant information of the current measurement.

Figure 11 shows the evolution of the current RMS value during the connection of different appliances. It is possible to observe the typical footprint of a washing machine (on the right) and a refrigerator (center) as well as the waveform in real time, where the presence of distortions owing to the effect of nonlinear loads is evident.

Figure 12 above plots the advanced view, where the full frequency spectrum of the current waveform is displayed up to approximately 7700 Hz. The peaks correspond to the odd harmonics. It can be seen that there is a strong presence of the 3rd, 5th, 7th, 9th, 11th, 13th, and 17th harmonics. All of these charts are responsive, allowing for zooming, panning, maximizing, and exporting data. At the bottom, the same visualization as in Figure 9 can be found.

### 3.3. Active and Reactive Power

**Figure 13:**
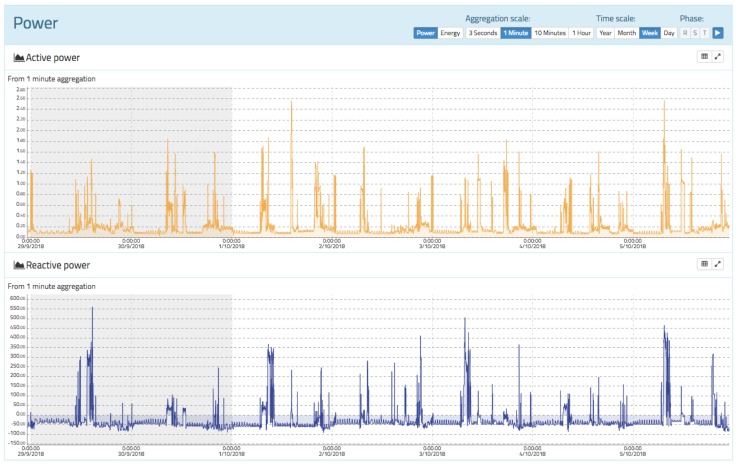
Active and reactive power view of oZm.

The power view enables the active and reactive power of the installation to be displayed (see Figure 13). The calculation of the active power is carried out using the well-known expression

(1) $$P=\frac{1}{N}\sum_{n=1}^{N}U_{n}I_{n}$$

while the reactive power is obtained as the result of the following product

(2) $$Q=U_{1}I_{1}\sin\varphi$$

i.e., the Budeanu approximation is not followed [49], so the power factor is calculated using the angle between the fundamental voltage and current through the FFT (see Section A2 regarding the use of varmeters in the presence of distorted waveforms from IEEE1459). However, the reactive power of the fundamental harmonic is computed. Other implementations are possible based on different proposals such as that defined by Czarnecki [50] and Castro-Nuñez [51] because of the open-source implementation of oZm.

Figure 13 shows the active power at the top and the reactive power at the bottom for a time span of 1 week with an aggregation level of 1 min. oZm supports aggregation scales ranging from 3 s to 1 h for web users, but up to 200 miliseconds for API users. On the left side, there is a shaded area corresponding to a weekend. In this case, only positive powers are shown because there is only consumption and no power generation. However, in the reactive power chart, both positive and negative values are observed because there are inductive (positive) and capacitive (negative) reactive power consumptions.

### 3.4. Frequency

The grid frequency is measured with great accuracy following the IEC 61000-4-30 standard. Thanks to the use of techniques based on digital filters and the use of the zero-crossing algorithm, a measurement with an error of less than 10 mHz is obtained in a range of ±15% of the nominal frequency. The zero-crossing algorithm was developed from scratch and takes into account the possible presence of interharmonics and noise. Firstly, five cascade biquad low-pass filters (tuned to the nominal frequency) are used to achieve a significant attenuation above the nominal frequency (50 or 60 Hz). Afterwards, we look for zero-crossing (change in the sign of the signal) at the output of the filter. The duration of each half cycle is stored in order to calculate the frequency, and when there are 20 sign changes the block is released for further processing by other modules of the system. The maximum block size is 10 cycles at nominal frequency ±15%. If all 20 half-cycles have not been found in that timeframe, the package is released creating a voltage event and labeling it with a flag. Likewise, if a cycle change is detected that is not where it is supposed to be (ahead or delayed ±15% to its position), we keep reading samples (if there is enough space), but the block is marked as a voltage event. Additionally, to detect sign changes, the signal must have a minimum amplitude. If it is below 10% of the nominal value, no cycles are searched, although blocks of the maximum size are read and later marked as interruptions. Using this technique, the system also avoids spectral leakage and picket fence effects because of the integer multiple of cycles computed in every batch of samples.

**Figure 14:**
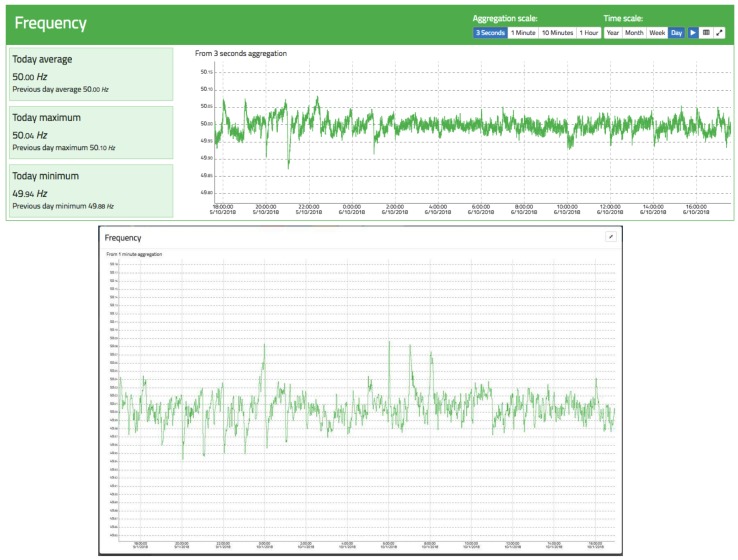
Frequency view of oZm. Top: general view. Bottom: maximized view.

Figure 14 shows an example of frequency tracking for a full day and an aggregation of 3 s. It can be seen that the average is exactly 50 Hz, oscillating around it with maximums and minimums of a few millihertz. Also noteworthy are the large oscillations at o’clock owing to the entry and exit of generator groups according to the Spanish electrical system’s time schedule, where some groups are disconnected/connected to adjust the generation to the demand. This causes accelerations/decelerations in the frequency according to the period of the day.

### 3.5. PQ Disturbances

**Figure 15:**
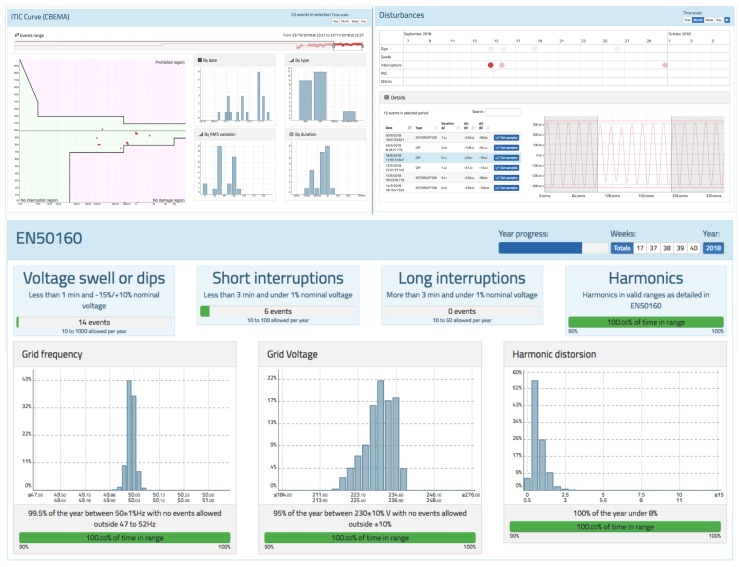
PQ events view of oZm. Top left: information technology industry council (ITIC) curve. Top right: disturbances table and viewer. Bottom: EN-50160 visualization and classification for voltage events.

One of the great features of oZm is its ability to measure some well-known PQ events. Several algorithms to monitor swells, dips, interruptions, and RVC following the international standards IEC61000-4-30 and EN-50160 have been implemented. The RMS voltage criterion, updated every half cycle, was used. For a comprehensive overview, three screens were implemented, as shown in Figure 15. The first screen is the ITIC/CBEMA curve, where a permitted zone, prohibited zone, and no-damage zone are defined. For each event detected, a new point is generated according to its magnitude in the voltage and time duration. Next to the curve, there are graphs showing statistics associated with these events.

The second screen is a timetable with points representing each type of event, as well as another table to select and visualize the waveform during the event. In this way, it is easy to observe what happened before and after the normal restoration of the voltage. Finally, following the EN-50160 standard, a visualization and statistics of the different types of events that occurred are presented. The number of events within the margins allowed by the standard are checked.

### 3.6. Active and Reactive Energy Stats

**Figure 16:**
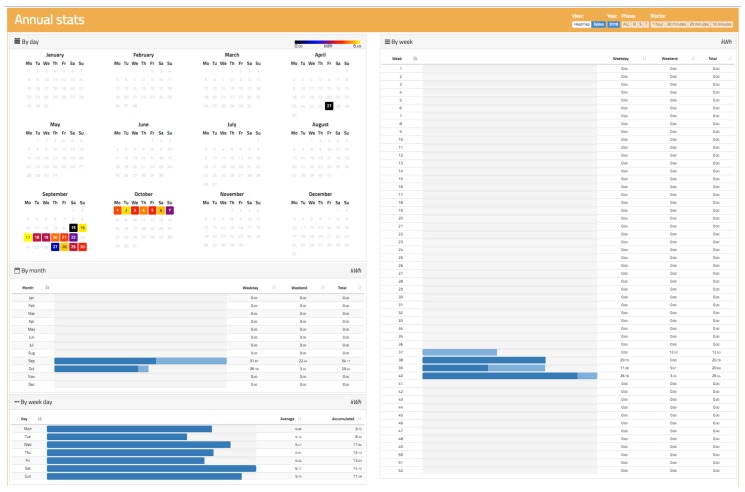
Active and reactive energy view of oZm.

**Figure 17:**
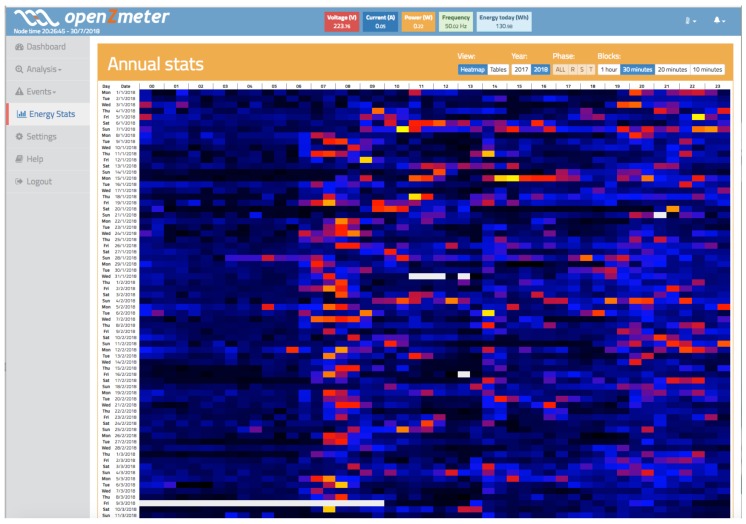
Active energy heat view of oZm.

An active and reactive energy measurement is carried out by adding the power measurement over time. Since the power was already analysed in previous sections, it is easy to obtain the accumulated values because this simply requires multiplication of the value of the power by time and determination of the consumption or generation of energy. For a better analysis, two visualizations were implemented, as shown in Figure 16 and Figure 17. Figure 16 shows a visualization more oriented to tables, where it is possible to analyse the energy habits depending on the year, month, week of the year, or day of the week. It is possible to distinguish between day and night periods. This is of great interest because many countries apply different pricing policies depending on whether energy is consumed during the day (where there is much more demand and the system is overloaded) than at night (where demand is much lower and energy prices are also lower).

Figure 17 shows a representation based on heat maps. Each coloured “pixel” represents the consumption made in a specific time span that can be adjusted between 10 min and 1 h so that there is more or less resolution according to the user’s interests. This visualization allows for a clear vision of the user’s consumption habits and provides a clear advantage over other representations when making decisions related to energy savings.

### 3.7. Comparison with Commercial Power Analyzer

In order to validate our proposal against existing commercial and certified devices, the features and operation of oZm have been compared with MyEBOX-1500 (MyEBOX from now on) power and energy analyzer from Circutor manufacturer, a commercial and certified device launched in 2016 which was designed for measurement and recording electrical parameters. According to the manufacturer, it incorporates the latest technology in portable measurement. Table 2 shows the main technical features and functions of oZm and Circutor MyEBOX. Moreover, an empirical study has been carried out to show how both devices measure, record, and visualize the electric data recorder in our university laboratory having some linear and non-linear loads.

**Figure 18:**
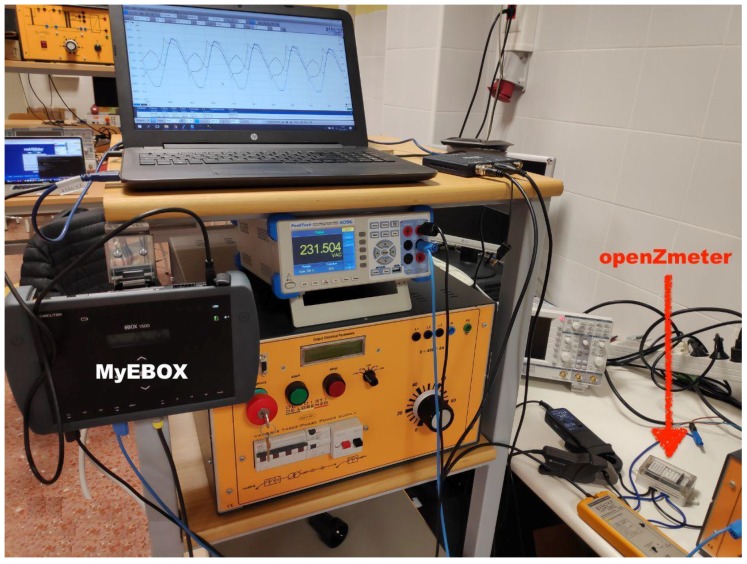
Setup for measurements with oZm and MyEBOX. Precission multimeter Peaktech 4096, Picotech 2000 oscilloscope, and Agilent power source also used.

**Figure 19:**
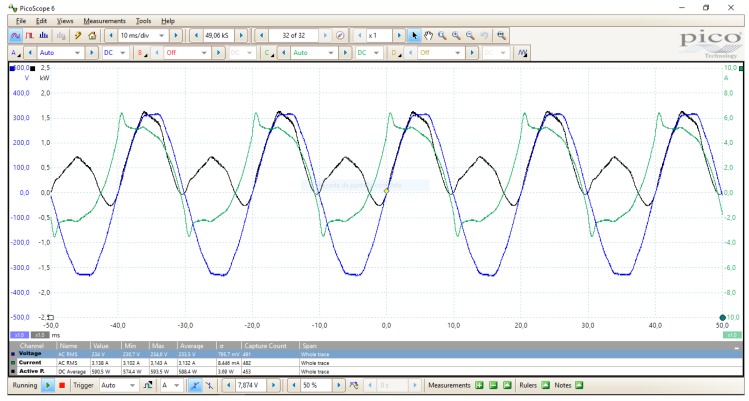
Non-linear load analysis with Picotech oscilloscope.

Three different scenarios have been configured and tested to perform measurements and compare results using oZm and MyEBOX (see Figure 18). First of all, a simple circuit consisting of a variable resistor 1017R from DeLorenzo and a 230VRMS/50 Hz sine wave voltage source from Agilent Technologies has been setup. Afterwards, the Agilent source was replaced by a mains supply from the laboratory grid. Finally, a non-linear load was connected to the laboratory mains. All scenarios have been monitored using a Picotech 2000 series oscilloscope with a high voltage active differential probe TA041. Figure 19 shows a capture of the measurement process for the non-linear load.

Table 3 shows a detailed comparison for the above scenarios. Voltage (V), current (I), active power (P), and frequency (F) have been measured during a 1 hour time span. Both oZm and MyEBOX show similar recorded values with minimal deviation. They also coincide with occasional readings using the Picotech oscilloscope and a precission voltimeter Peaktech 4096.

## 4. Conclusions

Nowadays, most electrical installations are monitored using commercial devices that are often expensive and difficult to handle for non-expert users. The main contribution of this research is the description of the oZm hardware, a low-cost, open-source, Internet-based multipurpose network analyzer and smart meter that has been designed, manufactured, and implemented for power quality and energy metering and submetering of electrical installations. The advanced design of oZm ensures reliable and accurate energy consumption and power quality measurements under a wide range of operating conditions in a single handy device. It can be used by anyone to analyze and visualize power consumption measurements and power quality events that comply with international standards such as IEC 61000-4-30 and EN-50160. Given its small size and large variety of current probes, it can be easily installed at any electrical installation, i.e., it can be used both as bulk metering or as metering/submetering for individual units or appliances. A comparison with commercial devices is also carried out. The characteristics and operation of oZm have been compared with MyEBOX-1500, an advanced commercial and certified power quality and energy meter. The results obtained in the laboratory show similar readings with minimal deviation. This comparison clearly shows that oZm is able to measure and record high precision RMS voltages, currents, frequency, and power quality events in different scenarios. Finally, other important contribution of the paper is to present and display a large number of measurements with an user-friendly interface that can be customized according the particular needs.

The acquired data is stored in a local PostgreSQL database, which is accessible using the network of the campus and a browser from any connected device. The main dashboard is accessible using the http protocol through an https secure layer. The potential benefits of oZm for domestic or industrial applications were demonstrated by the set of advanced measures it performs. The system proposed can be used for smart-grid studies and for advanced technique development such as non-intrusive load monitoring and machine learning applied to power quality. Moreover, the device is very easy to install and use by nonspecialized staff.

Although not all definitions of international standards IEC 61000-4-30 and EN-50160 were implemented, oZm is a constantly evolving project supported by the community. Research teams from several universities aim to complete and fulfill the requirements imposed by these standards. As a future work, we are now working on the development of a three-phase version of oZm.

## Figures and Tables

**Table 1:** Nomenclature.

Nomenclature	
AC	Alternating Current
ADC	Analog Digital Converter
AFE	Analog Front End
ARM	Advanced RISC Machine
CBEMA	Computer & Business Equipment Manufacturer’s Association
CRIO	Compact Reconfigurable i/o
CS	Comprensive Sensing
CSV	Comma-Separated Values
DC	Direct Current
DSP	Digital Signal Processing
FFT	Fast Fourier Transform
FPGA	Field Programmable Gate Array
HHT	Hilbert-Huang Transform
HTML	HyperText Markup Language
IC	Integrated Circuit
IEC	International Electrotechnical Commission
IoT	Internet of Things
ITIC	Information Technology Industry Council
JSON	JavaScript Object Notation
ML	Maximum Likelihood
MOSFET	Metal-Oxide-Semiconductor Field-Effect Transistor
NTP	Network Time Protocol
oZm	openZmeter
PCB	Printed Circuit Board
PNN	Probabilistic Neural Network
PQ	Power Quality
PQD	Power Quality Disturbances
RC	Resistor-Capacitor
RMS	Root Mean Square
RVC	Rapid Voltage Change
SVM	Support Vector Machine
THD	Total Harmonic Distortion

**Table 2:** List of main features of openZmeter (oZm) and MyEBOX.

	oZm	MyEBOX
Active Energy	Yes	Yes
Reactive Energy	Yes	Yes
Active Power	Yes	Yes
Reactive Power	Yes	Yes
Apparent Power	Yes	Yes
Frequency	Yes	Yes
RMS Voltage	Yes	Yes
RMS Current	Yes	Yes
Power Factor	Yes	Yes
Phase	Yes	Yes
4 Quadrants	Yes	Yes
Phasor	Yes	Yes
High Sampling Rate	Yes	Yes
Aggregated Intervals	Yes	Yes
Real-time alert system	Yes	No
Realtime Pricing	Yes	No
IEC61000/IEC61010	Yes	Yes
EN-50160	Yes	Yes
Voltage Events	Yes	Yes
ITIC/CBEMA	Yes	Yes
Zero Crossing	Yes	Yes
FFT	Yes	No
Harmonics	Yes	Yes
THD	Yes	Yes
Flickers	Yes	Yes
4G	Yes	No
Wi-Fi	Yes	Yes
Ethernet	Yes	No
API	Yes	No
HTML5 Interface	Yes	No
Telegram integration	Yes	No
Open Source	Yes	No

**Table 3:** Comparative test for oZm and MyEBOX under different scenarios.

		Sinusoidal Source and Resistor				Mains and Resistor				Mains and Non-Linear Load			
		Mean	Min	Max	StdDev	Mean	Min	Max	StdDev	Mean	Min	Max	StdDev
**V**	oZm	229.86	229.82	229.90	0.02	237.30	231.98	240.86	1.44	235.96	231.11	238.54	1.41
	MyEbox	229.37	229.33	229.39	0.01	237.46	232.07	241.04	1.45	236.14	230.24	238.87	1.49
**I**	oZm	2.24	2.23	2.26	0.01	2.30	2.25	2.33	0.01	3.17	3.12	3.20	0.02
	MyEbox	2.24	2.24	2.24	0.00	2.30	2.25	2.34	0.01	3.17	3.10	3.20	0.02
**P**	oZm	515.09	512.99	518.27	1.52	551.82	527.12	568.18	6.72	638.38	610.44	652.26	7.59
	MyEbox	513.08	513.00	514.00	0.27	546.78	522.00	563.00	6.60	617.81	586.00	633.00	7.94
**F**	oZm	50.00	50.00	50.00	0.00	49.99	49.92	50.04	0.02	50.00	49.96	50.04	0.02
	MyEbox	50.00	50.00	50.00	0.00	49.99	49.92	50.04	0.02	50.00	49.96	50.04	0.02
